# The interrupted effect of autophagic flux and lysosomal function induced by graphene oxide in p62-dependent apoptosis of F98 cells

**DOI:** 10.1186/s12951-020-00605-6

**Published:** 2020-03-18

**Authors:** Chao Zhang, Xiaoli Feng, Longwen He, Yaqing Zhang, Longquan Shao

**Affiliations:** 1 Stomatological Hospital, Southern Medical University (Guangdong Provincial Stomatological Hospital), Guangzhou, 510280 China; 2 Guangdong Provincial Key Laboratory of Construction and Detection in Tissue Engineering, Guangzhou, 510515 China

**Keywords:** Graphene oxide, Astrocyte, p62, Autophagy, Apoptosis

## Abstract

**Background:**

Graphene oxide (GO) nanoparticles (NPs) have been widely applied in various fields, especially in biomedical applications. Extensive studies have suggested that GO can pass through the blood–brain barrier (BBB) and induce abnormal autophagy and cytotoxicity in the central nervous system (CNS). However, the effect and specific mechanism of GO on astrocytes, the most abundant cells in the brain still has not been extensively investigated.

**Results:**

In this study, we systematically explored the toxicity and mechanism of GO exposure in the rat astroglioma-derived F98 cell line using molecular biological techniques (immunofluorescence staining, flow cytometry and Western blot) at the subcellular level and the signaling pathway level. Cells exposed to GO exhibited decreased cell viability and increased lactate dehydrogenase (LDH) release in a concentration- and time-dependent manner. GO-induced autophagy was evidenced by transmission electron microscopy (TEM) and immunofluorescence staining. Western blots showed that LC3II/I and p62 were upregulated and PI3K/Akt/mTOR was downregulated. Detection of lysosomal acidity and cathepsin B activity assay indicated the impairment of lysosomal function. Annexin V-FITC-PI detection showed the occurrence of apoptosis after GO exposure. The decrease in mitochondrial membrane potential (MMP) with an accompanying upregulation of cleaved caspase-3 and Bax/Bcl-2 further suggested that endogenous signaling pathways were involved in GO-induced apoptosis.

**Conclusion:**

The exposure of F98 cells to GO can elicit concentration- and time-dependent toxicological effects. Additionally, increased autophagic response can be triggered after GO treatment and that the blocking of autophagy flux plays a vital role in GO cytotoxicity, which was determined to be related to dysfunction of lysosomal degradation. Importantly, the abnormal accumulation of autophagic substrate p62 protein can induce capase-3-mediated apoptosis. Inhibition of abnormal accumulation of autophagic cargo could alleviate the occurrence of GO-induced apoptosis in F98 cells.

## Background

Graphene oxide (GO) nanoparticles (NPs) have been widely used in biomedical fields due to their physical and chemical properties, which make them useful for applications in as drug delivery [1, 2], tumor photothermal therapy [3–5], bioimaging [6], tissue engineering [7, 8], antimicrobial agents [9, 10], biosensors [11–14]. At the same time, the risk of human contact has increased dramatically. A growing number of studies have reported that NPs can penetrate the blood–brain barrier (BBB) or enter brain tissues through nerve uptake, leading to potential dangers of the central nervous system (CNS) [15, 16].

Astrocytes are the most abundant and widely distributed predominant cell group in the mammalian CNS, which performs critical functions vital to CNS physiology [17]. The formation of the BBB by astrocytes and endotheliocytes affects the passage of NPs into the CNS, which participates in the termination and recycling of neurotransmitters through the glutamate–glutamine cycle and mediates the toxicity of neurons to NPs via the secretion of a series of cytokines and inflammatory cytokines [18, 19]. Therefore, studying the toxicity of astrocytes to NPs is an important part of the CNS toxicity to NPs [20]. Studies have shown that the uptake and internalization of titanium dioxide NPs can inhibit proliferation, induce the depolymerization of F-actin morphological changes, and lead to apoptosis in glial cells [21]. Exposure to silver NPs or zinc oxide NPs can induce oxidative stress and apoptosis of astrocytes [22, 23]. In addition, toxic effects on astrocytes are related to many neurodegenerative diseases, such as Alzheimer’s disease, Parkinson’s disease, Huntington’s disease, ischemic stroke and epilepsy [24, 25]. Considering the crucial role of astrocytes and the great potential application of GO in the CNS, studying the effect and specific mechanism of GO on astrocytes is urgently required.

Autophagy, namely, macroautophagy in mammals, is a dynamic and multistep process that includes the formation of autophagosomes that engulf intracellular components, fusion between autophagosomes and lysosomes to form autolysosomes and, finally, degradation of the intracellular content in lysosomes [26]. The entire process of autophagy is also called autophagic flux. Microtubule-associated protein 1 light chain 3 (LC3) is a marker of autophagy and has been confirmed to be involved in the entire process of autophagy. During autophagy, cytosolic LC3 (LC3I) hydrolyzes a small segment of polypeptide and converts to a phosphatidylethanolamine (PE)-conjugated form (LC3II), which functions as an integral membrane protein of autophagosomal membranes [27, 28]. The P62 protein is a ubiquitin-LC3-binding protein. In the late stage of the development of autophagy flux, p62 can mediate the formation of a complex between the ubiquitin substrate and LC3II and finally enter the autolysosome for degradation [29]. It was reported that after astrocytoma cells or primary astrocytes were exposed to amine-modified polystyrene NPs, apoptotic reactions and lysosomal acidification were observed [30]. In addition, PC12, a neuronal cell model, could induce apoptosis after exposure to GO by damaging autophagic flux [31]. Some NPs can cause autophagic flux perturbation and lysosomal dysfunction, leading to toxicological consequences [32]. However, it is not clear how GO affects the process and signaling pathways of autophagy in astrocytes, and the specific relationship between autophagy and apoptosis in the participation of GO remains unclear.

The purpose of this study was to investigate the effects of GO on cell viability, autophagy flux, lysosomal function and apoptosis in a rat astroglioma-derived F98 cell line. We used immunofluorescence staining, flow cytometry, Western blot and other molecular biological techniques to study the toxic mechanism at the subcellular level and the signaling pathway level. This study focuses on the toxicity of F98 cells to GO to fill the current gap regarding the effects of GO on astrocytes and to provide insight into the safe application of GO in the CNS.

## Methods

### Reagents and antibodies

GO nanosheet was obtained from Sigma-Aldrich (Shanghai, China). Dulbecco’s modified Eagle’s medium (DMEM), fetal bovine serum (FBS), streptomycin–penicillin and EDTA/trypsin were purchased from Thermo Fisher Scientific (Shanghai, China). An LDH-Cytotoxicity Assay Kit and a bicinchoninic acid (BCA) protein assay kit were from Beyotime (Nanjing, China). A cell counting kit-8 (CCK-8) test was purchased from Dojindo Molecular Technologies (Shanghai, China). Rapamycin and carbonyl cyanide 3-chlorophenylhydrazone; carbonyl cyanide 3-chlorophenylhydrazone; carbonyl cyanide m-chlorophenylhydrazone (CCCP) were from MCE (Shanghai, China). 2-(4-amidinophenyl)-6-indolecarbamidine dihydrochloride (DAPI) staining solution was obtained from Leagene Biotechnology (Beijing, China). 2.5% glutaraldehyde for electron microscopy was obtained from Nacalai Tesque, INC (Japan). Lyso-Tracker Red and RIPA lysate buffer were obtained from Solarbio Life Sciences (Beijing, China). A human cathepsin B activity assay kit was purchased from Cusabio (Wuhan, China). An Annexin V-FITC-PI Apoptosis Kit and a Mitochondrial Membrane Potential (MMP) assay kit (JC-1) were obtained from KeyGEN BioTECH (Jiangsu, China). Rapamycin was purchased from HefeiAnqi Biotech Co., Ltd. (Hefei, China). Halt protease inhibitor and phosphatase inhibitor cocktail was from La Roche Ltd. (USA). Polyvinylidene fluoride (PVDF) membranes were purchased from R&D Systems (USA). Primary antibodies, including antibodies for LC3I/II, p62, PI3K, Akt/p-Akt, mTOR/p-mTOR, Bax, Bcl-2, caspase-3, and cleaved caspase-3, and horseradish peroxidase-conjugated (HRP) secondary antibodies were obtained from Abcam (Shanghai, China). Chemiluminescence substrate was obtained from EMD Millipore (Massachusetts, USA).

### Characterization of GO NPs

The microstructure of GO was observed by scanning electron microscopy (SEM; Hitachi Ltd., Japan). Moreover, an atomic force microscope (AFM; Agilent Inc., USA) and a transmission electron microscope (TEM; Hitachi Ltd, Japan) for the original characterization of GO.

The functional groups and molecular structure of GO were determined by Raman spectroscopy (Renishaw, UK) and Fourier-transform infrared spectroscopy (FTIR) (Thermo Fisher Inc., USA). Basic chemical bonds and element content of GO were detected using X-ray photoelectron spectroscopy (XPS; Kratos, Japan).

GO nanosheets were sonicated using an ultrasonic processor (Biosafer, China) in pure water and cell culture medium to obtain uniformly dispersed suspension. The hydrodynamic size and zeta potentials of GO NPs in solution with uniform dispersion were measured using a Nano Zetasizer (Malvern Panalytical Ltd., England).

### Cell culture and preparation of GO suspension

The rat astroglioma-derived F98 cell line (ATCC CRL-2397) was purchased from Zhongqiao Xinzhou Co., Ltd. (Shanghai, China). After recovery from liquid nitrogen, F98 was cultured in DMEM containing 10% FBS and 1% streptomycin-penicillin and incubated in 95% humidified air with 5% carbon dioxide at 37 °C to obtain a culture density of 1000 cm^−2^. During the culture process, the growth status and cellular morphology were observed using an ICX41 inverted biomicroscope (Sunny Optical Ltd, China). The cell culture medium was replaced every other day, and the average subculture time was 3–4 days.

A GO suspension with a concentration of 1 mg/mL was obtained in advance as the stock solution. After irradiation with ^60^Co, GO nanosheets were sonicated for 1 h 8-times (60 Hz) by an ultrasonic processor. Before each administration, the storage solution of GO was diluted to different concentrations with complete medium supplemented with 10% FBS and then ultrasonicated again for 30 min with a high-power ultrasonic crusher.

### Cell viability assay

F98 cells were first inoculated in 96-well plates (Corning, USA) at a density of 5 × 10^3^ cells/well and cultured in a 37 °C incubator for 24 h prior to GO treatment. Then, the cells were exposed to fresh GO suspension at final concentrations of 10, 20, 40, 50, 60, 80, and 100 μg/mL, while the control group was left untreated. After 6, 12 and 24 h of treatment, cell proliferation activity was evaluated by the CCK-8 test according to the supplier’s protocol.

Cellular viability was further estimated by measuring the LDH release from injured cells. Briefly, the F98 cells were inoculated in 96-well plates (5 × 10^3^ cells/well) and cocultured with GO suspension at different concentrations (20, 40, 50, 60 and 80 μg/mL) for 6 and 24 h. Wells containing only culture medium without cells (background blank control) and wells containing cells without GO (sample control) were used as negative controls. For the positive control, the maximum release of LDH was triggered by LDH release agent. After treatment, all supernatants were transferred to new 96-well plates and assessed by the LDH assay kit according to the supplier’s protocol. At a wavelength of 490 nm, the absorbance was analyzed using a full-spectrum microplate reader (Reagen, China).

On the basis of the CCK-8 cell viability test, 40, 60 and 80 μg/mL GO were selected, and rapamycin (100 nM) was added for the same amount of time in the rescue experiment. Similarly, we measured cell viability by CCK-8 and LDH kits.

### SEM and TEM observation

F98 cells were seeded on coverslips in 12-well plates (5 × 10^4^ cells/well) and cocultured with or without GO (60 μg/mL) for 24 h. Thereafter, the cells were fasted overnight with 2.5% glutaraldehyde at 4 °C and washed with PBS. Subsequently, 1% osmium tetroxide was added to treat the cells at 4 °C for 1 h. Finally, the cell coverslips were dehydrated with graded ethanol and gold sputtered. The cells were observed and photographed with a Hitachi S-3400N SEM.

For TEM observations, F98 cells were seeded in 6-well plates (10^5^ cells/well) and then cocultured with or without GO (60 μg/mL) for 24 h. The cells were washed with PBS, harvested with trypsin, centrifuged at 800 rpm for 5 min, and then fasted with 2.5% glutaraldehyde for 50 min at room temperature and for 3 h at 4 ℃. After dehydration with a graded series of ethanol, the cells were embedded with epoxy resin at 90 °C for 3 days. Finally, ultrathin sections  (70 nm) were dyed with uranyl acetate-lead citrate and then observed and photographed by TEM.

### Detection of autophagy flux formation by immunofluorescence staining

As previously described, F98 cells were seeded on glass coverslips and co-exposed with or without GO (60 μg/mL) for 24 h. Cells were also treated for the same amount of time with rapamycin (100 nM) as the positive control. Thereafter, the cells were washed with PBS and fasted with 4% paraformaldehyde for 9 min. Then, the cells were blocked for 1 h, cocultured with the primary antibody at 4 °C overnight, and finally cocultured with the secondary antibody for 45 min at room temperature. The cells were dyed with DAPI staining solution at room temperature for 5 min. Images were immediately observed by a BX63-AFM microscope (Olympus, Japan). FITC and DAPI excitation/emission filters were selected for photography.

### Detection of lysosomal acidity

F98 cells were seeded and treated with 40, 60 and 80 μg/mL GO for 24 h. After incubation, Lyso-Tracker Red was diluted to a working concentration with complete medium at a ratio of 1:16,000 and then cultured with cells at 37 °C for 1 h. The cells were then washed with PBS. After removing the excess water with filter paper, the cells were inverted on a carrier plate, dripped with anti-fluorescence quenching tablet sealer, and observed by a BX63-AFM microscope. A FITC excitation/emission filter (Ex = 495 nm, Em = 515 nm) was selected for photography. To quantitatively evaluate the strength of FITC fluorescence, cells were harvested with trypsin after the same treatment and then resuspended in PBS and instantly analyzed by flow cytometry (BD Biosciences, USA).

### Cathepsin B activity assay

Cells were inoculated in 9 cm culture dishes at a density of 9 × 10^5^ cells/dish. Similarly, F98 cells were treated with 40, 60 and 80 μg/mL GO for 24 h. The cells were subsequently washed with precooled PBS and harvested with trypsin. After two cycles of repeated freezing and thawing, the debris and cell fragments were centrifuged at 5000 rcf for 5 min to collect the supernatant liquid, and cathepsin B activity was detected according to the supplier’s protocol.

### Annexin V-FITC-PI assay

F98 cells were plated on 6-well plates (2 × 10^5^ cells/well) and treated with 40, 60 and 80 μg/mL GO for 24 h. In the rescue experiment, 60 μg/mL GO and 60 μg/mL GO-100 nM rapamycin were included as the experimental groups. A blank control group and a rapamycin control group were also included. The old culture medium was abandoned, and the cells were washed with PBS. After that, the cells were harvested with trypsin and washed again with PBS. Then, 500 μL of binding buffer was added to the resuspended cells, and 5 μL of Annexin V-FITC and 5 μL of propidium iodide (PI) were added in turn. After incubation in the dark at room temperature for 15 min, the percentages of apoptotic cells were immediately detected by flow cytometry.

### Nuclear staining with DAPI

Cells were cultured overnight on polylysine-coated dishes and then treated with 0, 40, 60 and 80 μg/mL GO for 24 h. Cells were treated separately with CCCP (50 μM) for 30 min as a positive control. After incubation, the cells were harvested with trypsin, subsequently washed with PBS, fasted with 4% paraformaldehyde for 30 min, and then labeled with DAPI staining solution for 10 min. Changes in the nuclei were instantly observed by a BX63-AFM microscope. A DAPI excitation/emission filter (Ex = 364 nm, Em = 454 nm) was selected for photography.

### Assessment of MMP

Similarly, after 24 h of exposure to 40, 60 and 80 μg/mL GO, the cells were washed with PBS and incubated at 37 °C for 20 min with 5 µg/mL JC-1, a fluorescent carbocyanine dyestuff widely used to detect MMP. The cells were then washed and resuspended in JC-1 assay buffer (1×), and red-green fluorescence was immediately detected by flow cytometry.

### Western blots

After 24 h of treatment with 40, 60 and 80 μg/mL GO, the cells were washed with PBS, and RIPA lysate buffer containing protease inhibitor and phosphatase inhibitor cocktail were prepared to digest the cells. Cell granules and lysates were collected and centrifuged at 12,000 rpm for 30 min. The supernatant was saved, and the total protein content was measured by a BCA protein assay kit. The newly extracted protein was used in 10% SDS-PAGE (sodium dodecyl sulfate polyacrylamide gel electrophoresis) and then transferred to polyvinylidene fluoride (PVDF) membranes. Subsequently, the membranes were blocked for an hour and then incubated overnight with primary antibody at 4 °C. The primary antibodies included LC3I/II, p62, PI3K, Akt/p-Akt, mTOR/p-mTOR, Bax, Bcl-2, caspase-3, and cleaved caspase-3. Thereafter, the membranes were incubated with HRP-conjugated secondary antibody at room temperature for an hour. GAPDH (glyceraldehyde-3-phosphate dehydrogenase) was designated as a loading control and showed no significant differences among groups. Finally, the protein bands were activated by chemiluminescent substrate and visualized by an enhanced chemiluminescence (CL) system (Tanon Science & Technology Co., Ltd, China). The band intensities were quantified using ImageJ.

In the rescue experiment, 60 μg/mL GO and 60 μg/mL GO-100 nM rapamycin were included as the experimental groups. A blank control group and a rapamycin control group were also included. The primary antibodies were LC3I/II, p62, Bax, Bcl-2, caspase-3, and cleaved caspase-3, and the same operational steps were used as described above.

### Statistical analysis

The results are expressed as the mean ± standard deviation (SD) values of three independent experiments. All analyses were carried out using GraphPad Prism (USA) with unpaired Student’s t-test or one-way ANOVA. The data were considered to be statistically significant when the probability (p) < 0.05.

## Results

### Characterization of GO

**Fig. 1:**
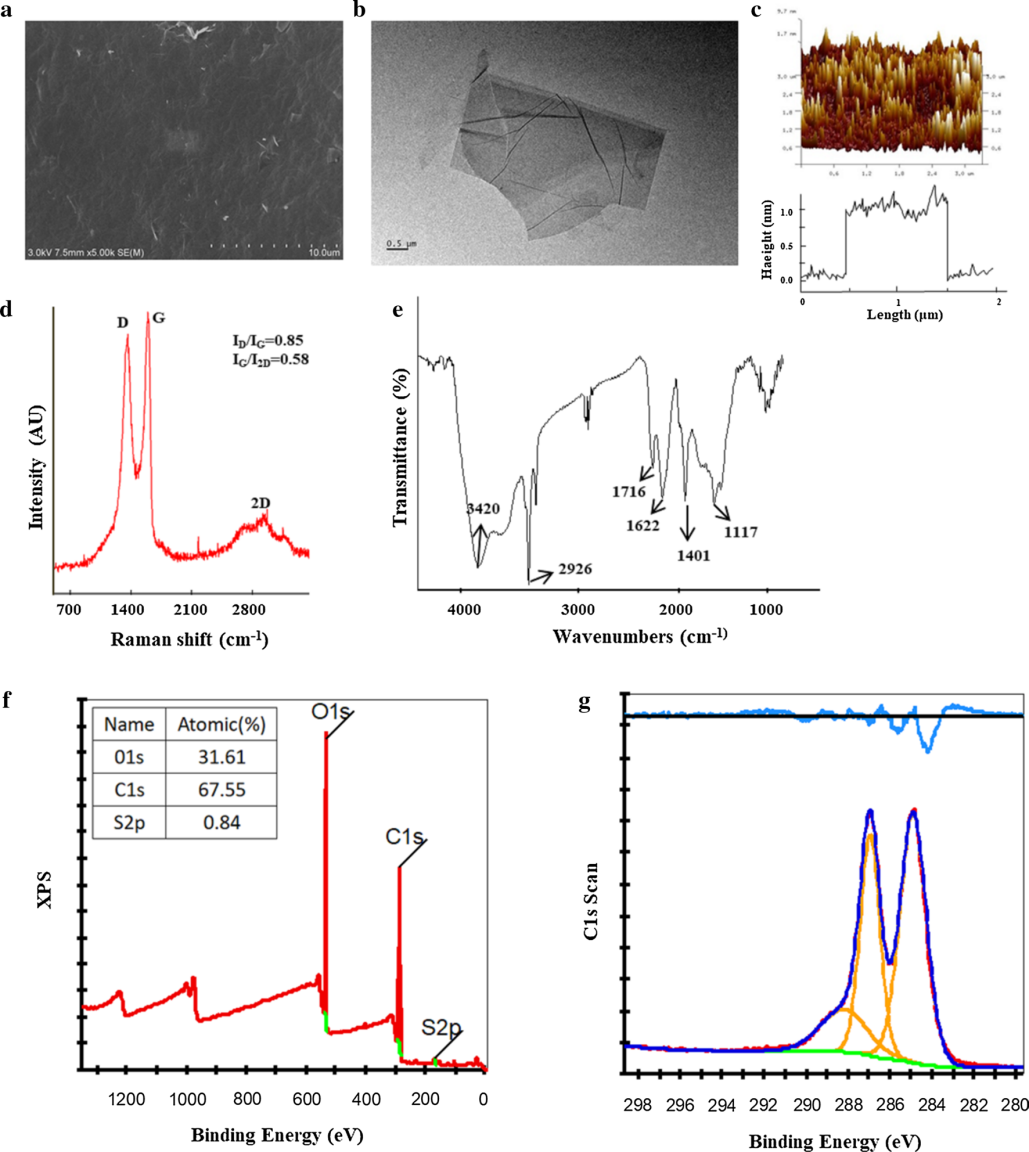
Characterization of GO nanomaterials. **a**–**c** SEM, TEM and AFM images of GO; **d** identification of Raman spectroscopy; **e** identification of GO functional groups by FTIR; **f**, **g** authentication of XPS to investigate the chemical composition of surfaces of GO

SEM showed that GO is a two-dimensional single-layer material with sharp and irregular edges and that the lateral size distribution varies from 50 to 800 nm (Fig. 1a). TEM further showed that large pieces of GO were flaky with a thin and wrinkled surface, easy to curl (Fig. 1b). The results of AFM (Fig. 1c) are consistent with SEM and TEM, and the data analysis shows that the average thickness of GO used in this experiment was approximately 1.0 nm.

Raman spectroscopy revealed three main peaks of GO: a D peak (1368 cm^−1^), a G peak (1602 cm^−1^) and 2D peaks at 2761.46 and 2769.17 cm^−1^ with symmetric line shapes (Fig. 1d). The I_D_/I_G_ strength ratio was 0.85, which indicates that the molecular conformation of graphene changed after oxidation, i.e., via lattice distortion. The I_G_/I_2D_ strength ratio was 0.58 (< 0.7), which confirms a single-layered graphene. The FTIR spectra show that the 3420 cm^−1^ and 1401 cm^−1^ positions correspond to the O–H (hydroxyl) vibration absorption peak and deformation absorption peak, respectively (Fig. 1e). The C=O (carboxyl) stretching vibration wave is located at 1716 cm^−1^. The peak for C=C in the hybrid form of sp^2^ is located at 1622 cm^−1^, and the C–O (alkoxy) stretching vibration wave is located at 1117 cm^−1^. Furthermore, the results of the full-spectrum analysis showed that the C, O and S content in the GO samples was 67.55%, 31.61% and 0.84%, which further confirmed the existence of oxygen groups in GO (Fig. 1f). XPS also revealed the basic chemical bonds of GO, including C–O (287.1 eV), C=O (288.2 eV), O–C=O (289.3 eV), and C=C (284.7 eV) (Fig. 1g).

### Concentration and exposure time-dependent cytotoxicity induced by GO

**Fig. 2:**
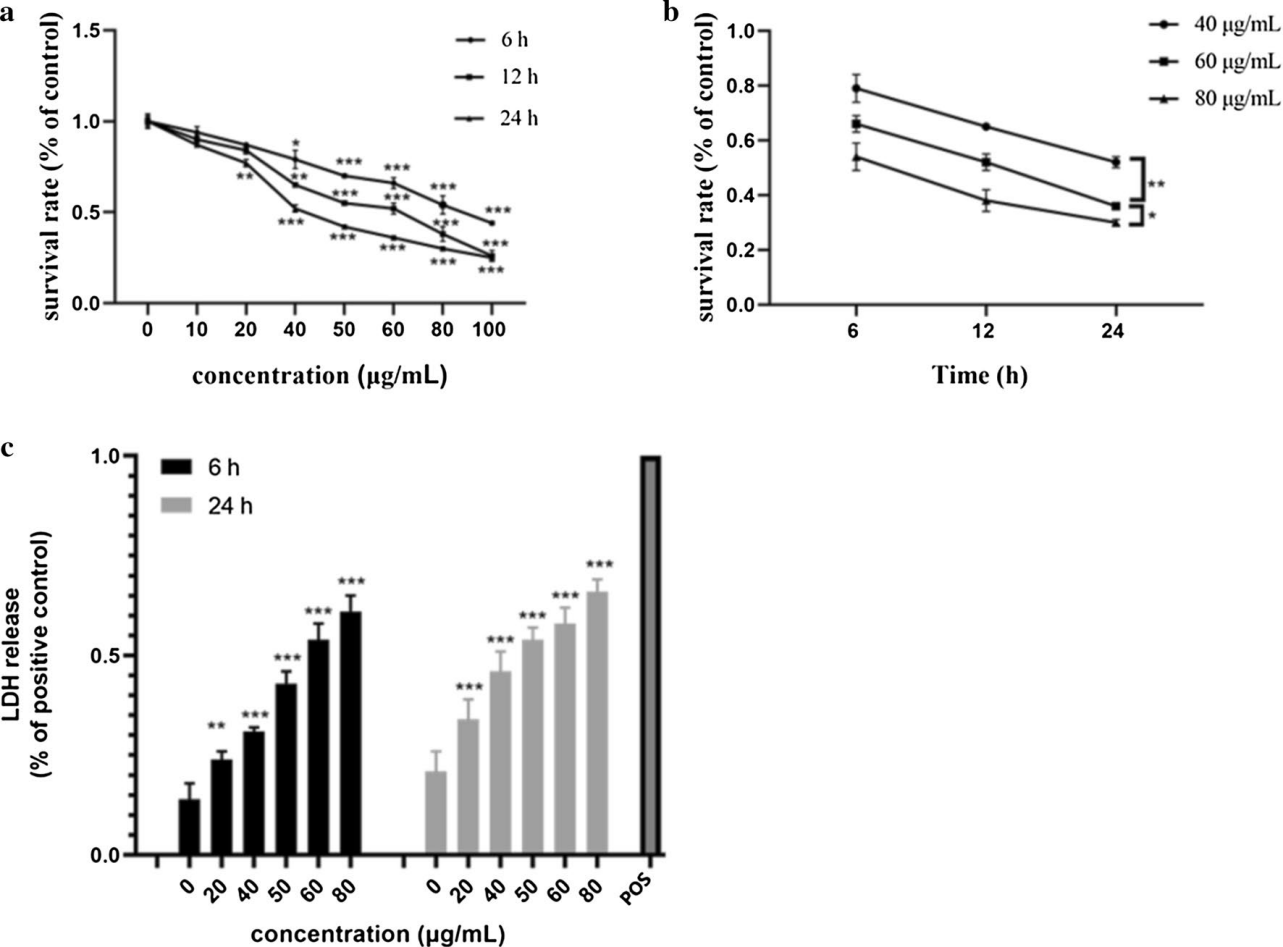
Concentration- and time-dependent cytotoxicity of GO in F98 cells. **a**, **b** Cells were exposed to GO at different concentrations (10, 20, 40, 50, 60, 80 and 100 µg/mL) for different amounts of time (6 h, 12 h and 24 h), and cell viability was assayed by CCK-8 assay. **c** Detection of LDH release in F98 cells treated with different concentrations of GO (20, 40, 50, 60 and 80 µg/mL) for 6 h and 24 h. The positive control (POS) corresponded to 100% LDH release. All data are expressed as the mean ± standard deviation (SD) of three independent experiments. *p < 0.05, **p < 0.01 and ***p < 0.001 vs. the control group

The cell viability evaluated by the CCK-8 assay showed a significant decrease at 80 μg/mL after 6 h of exposure compared with untreated cells (Fig. 2a). Nearly 50% of cell viability was observed after 12 h of treatment with 60 μg/mL GO, while a similar effect was observed after 24 h of exposure to 40 μg/mL GO. The results showed that GO had a significant concentration dependence on the proliferation toxicity of cells, which was consistent with the dose-dependent neurotoxicity of GO in rat pheochromocytoma PC12 cells [33]. We further studied the effect of increasing the exposure time of GO on cell viability. According to the known 50% lethal dose (LD50) from CCK-8, three crucial concentrations of GO (40, 60, 80 μg/mL) were selected to observe the cytotoxicity after 6 h, 12 h and 24 h, and then, the cell viability was detected. The results showed that the activity of cells exposed to GO for 24 h was markedly lower than that of cells treated with GO for 6 h, which revealed significant time-dependent toxicity (Fig. 2b). Interestingly, for 80 μg/mL GO, the cell viability was 38% at 12 h and 30% at 24 h, indicating that compared with prolonged culture time, the high concentration of GO had a stronger effect on cell viability.

LDH release is an important indicator of cell membrane damage and cytotoxicity. Compared with the positive control, the LDH level in untreated cells was 21%, while the LDH level in cells exposed to GO at a concentration of 80 μg/mL was 66% after 24 h (Fig. 2c). Moreover, the release of LDH at 24 h was always higher than that at 6 h, which was reflected in several concentrations. These results indicated that GO dose- and time-dependently increased the leakage of LDH.

### Cellular adhesion and uptake of GO

**Fig. 3:**
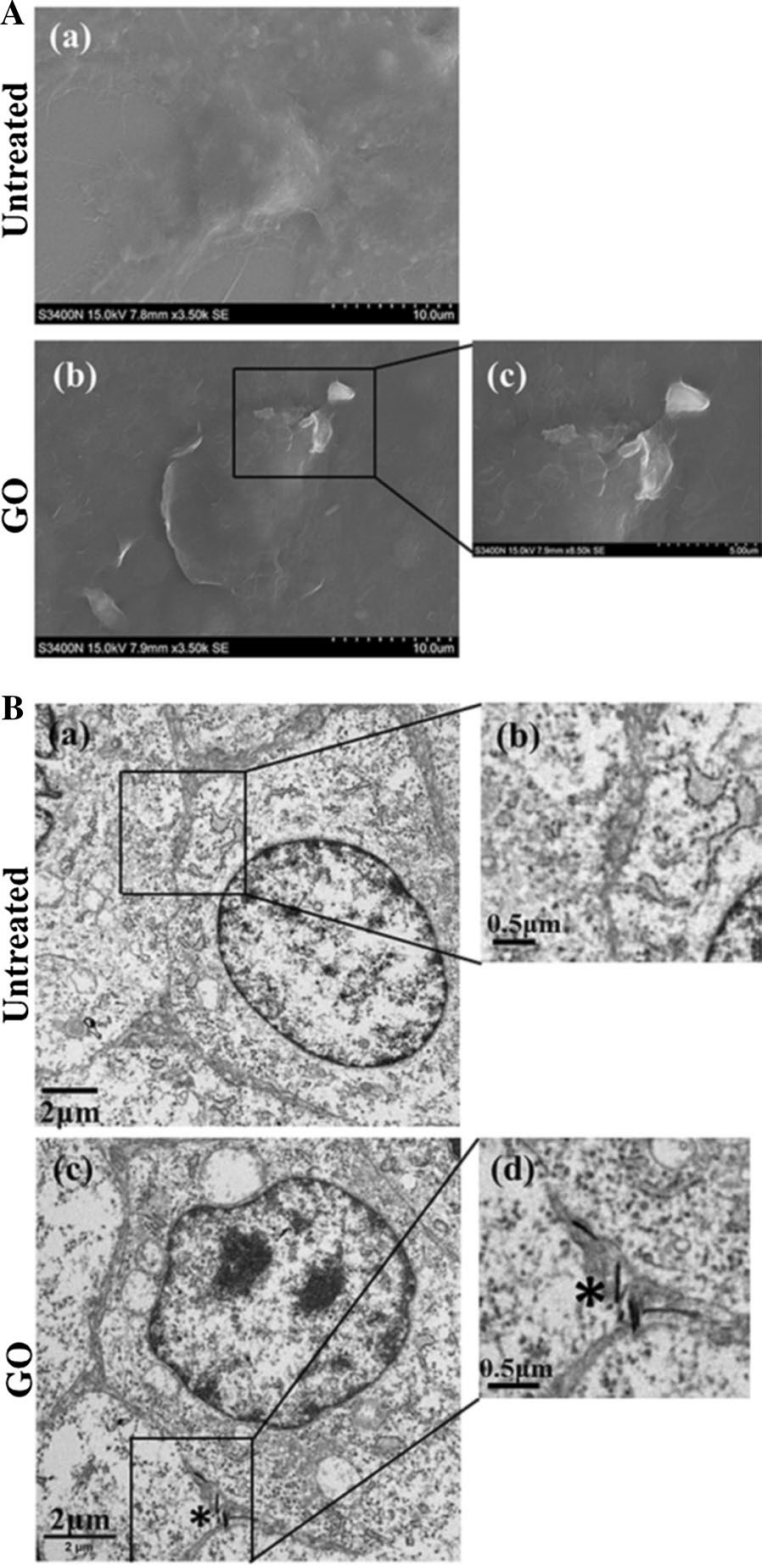
The uptake and adhension of GO adhering to F98 cells. Identification of SEM (**a**) and TEM (**b**) imagines of F98 cells after treated with GO at the concentration of 60 µg/mL for 24 h. The magnified images of the boxed area are shown on the right. *Indicates graphene oxide

SEM observation showed that the cells in the control group were star-shaped with a smooth surface, clean background and no obvious impurities. After GO treatment, there was no significant change in the overall morphology of the cells, while there was a sheet of GO adherence on the surface and a large number of GO fragments in the background (Fig. 3a). The TEM results showed that the cell membrane exposed to GO was sunken inward, and lamellar GO was observed close to and even warped into the cell (Fig. 3b). SEM and TEM observations confirmed that GO interacts with the cell membrane to induce cytotoxicity.

### Autophagy is triggered following GO exposure

**Fig. 4:**
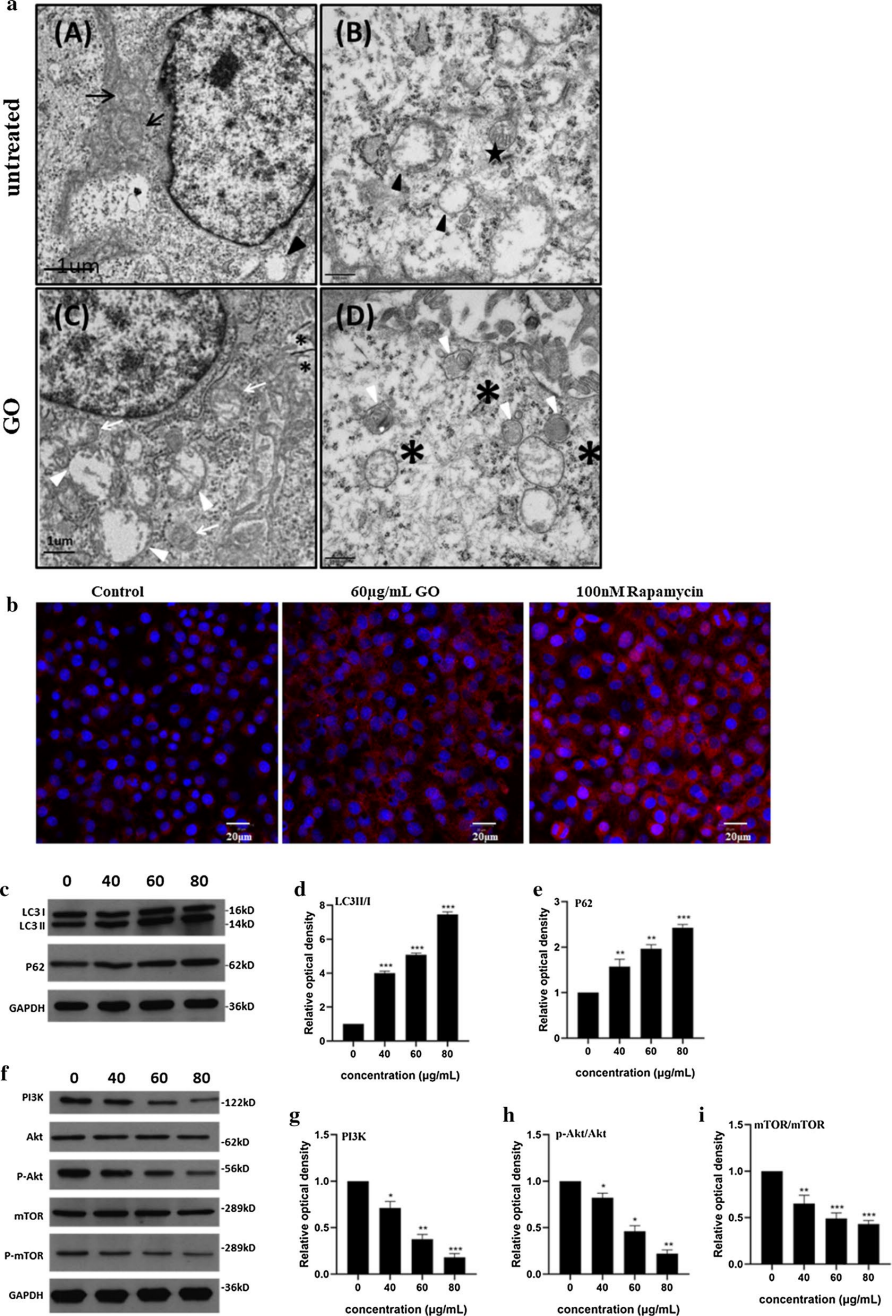
Go triggers autophagy in F98 cells. **a** Identification of TEM imagines of F98 cells after treated with GO at the concentration of 60 µg/mL for 24 h. Black arrowhead and black arrow indicate autophagosome and mitochondria, respectively. Black pentagon indicates initial autophagic vacuoles (AVi), containing a mitochondrion, endoplasmic reticulum membranes and ribosomes. Autophagic vacuoles (white arrowhead), mitochondria swelling and cristae vague (white arrows) as well as GO materials (*) were denoted; **b** F98 cells were treated with 60 µg/mL GO for 24 h. In addition, autophagy activator rapamycin (8 nM) was added for the same time as a positive control. The imagines of LC3 fluorescent labeling were captured under a fluorescence microscope; **c**–**e** the expression of P62 and LC3 II/I in F98 cells after treating with 40, 60 and 80 µg/mL GO for 24 h; **f**–**i** The expression of PI3K, Akt/p-Akt and mTOR/p-mTOR in F98 cells after exposing to 40, 60 and 80 µg/mL GO for 24 h. All datas expressed as mean ±  SD in three independent experiment results. *p  < 0.05, **p  <  0.01 and ***p  < 0.001 vs. control group

TEM was used to further observe the distribution of GO in cells and its potential effect. The results showed that after GO treatment, extensive cytoplasmic vacuolization, including vacuolated mitochondria, could be observed in cells (Fig. 4a). In addition, GO is encapsulated by intracellular vesicles, such as autolysosomes and lipid vesicles. GO is a type of bad stimulation after internalization into cells, resulting in intracellular homeostasis imbalance and even autophagy induction. Studies have shown that autophagy can be triggered after GO treatment and that the blocking of autophagy flux plays a vital role in GO cytotoxicity [34, 35].

Autophagy is a unique cellular process for degrading long-lived proteins, damaged organelles and foreign bodies via the lysosomal degradation pathway. There are many molecules and proteins involved in autophagy, among which LC3 has been shown to be involved in the entire process of autophagy formation, and the accumulation of LC3II is a well-known sign of autophagy. To assay autophagic activation, LC3 was stained by single fluorescence, and the fluorescence intensity was observed under a fluorescence microscope, one of which was equivalent to an autophagosome [36]. The results showed no obvious fluorescent spots in the cytoplasm of untreated cells (Fig. 4b). After treatment with 60 μg/mL GO for 24 h, a large number of fluorescent spots appeared in the cytoplasm. Rapamycin treatment, as a positive control, also induced a large number of autophagic fluorescent spots. The red fluorescent expression of cells in the positive control group was stronger than that in the GO-treated group, which showed that rapamycin enhanced autophagy [37]. The fluorescence microscopy results further confirmed that GO triggered autophagy in F98 cells, which was consistent with the TEM results.

The Western blotting results (Fig. 4c) showed that after GO treatment for 24 h, the ratio of LC3II/I was markedly upregulated (Fig. 4d), and the expression of p62 was also upregulated (Fig. 4e). It is worth noting that cells exposed to 80 μg/mL GO exhibited more obvious upregulation than those exposed to 60 μg/mL and 40 μg/mL GO. With increasing GO concentration, LC3II/I and p62 expression increased more significantly compared with the control group, indicating that GO acts on F98 cells in a dose-dependent manner.

The expression of PI3K/Akt/mTOR, a main upstream signaling pathway of autophagy, was further evaluated by Western blot analysis. The results (Fig. 4f) showed that PI3K (Fig. 4g), p-Akt (Fig. 4h) and p-mTOR (Fig. 4i) were downregulated after 24 h of GO treatment, indicating that PI3K/Akt/mTOR signaling pathway function was inhibited during GO-induced autophagy. In addition, analysis of the protein band densities showed that higher GO dosages corresponded to more significant decreases in protein expression level, with a certain concentration dependence.

### GO induced lysosomal dysfunction in F98 cells

**Fig. 5:**
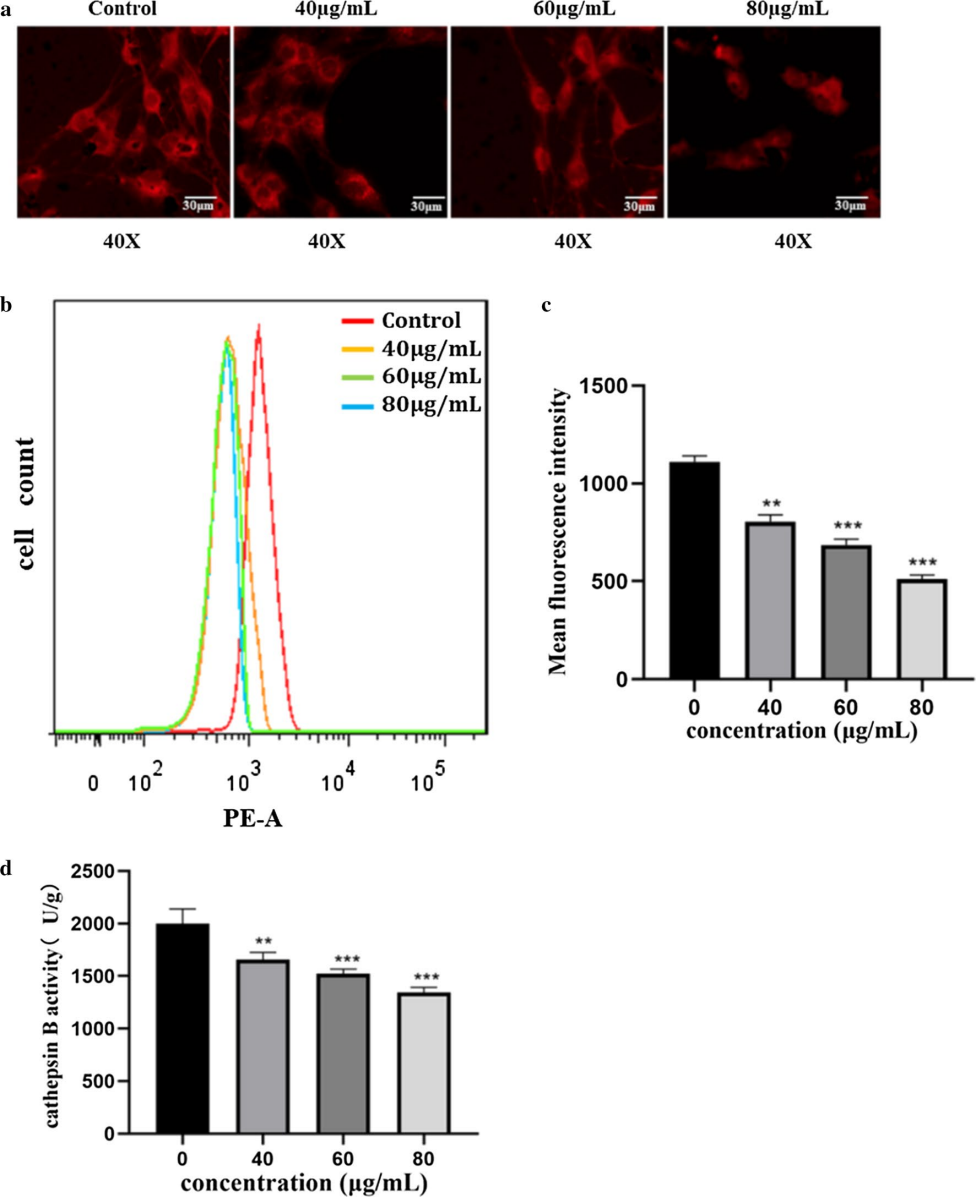
GO induced lysosomal dysfunction in F98 cells. **a** F98 cells were exposed to 40, 60 and 80 μg/mL GO for 24 h, and then incubated with 1 μmol/L Lyso-Tracker Red. Representative imagines were photographed under a fluorescent microscope. **b**, **c** Flow cytometry was used to further detect Lyso-Tracker Red test for quantitative analysis of fluorescence images. **d** Determination of cathepsin B activity in F98 cells treated with 40, 60 and 80 μg/mL GO for 24 h. All datas expressed as mean ± SD in three independent experiment results. *p < 0.05, **p < 0.01 and ***p < 0.001 vs. control group

Lyso-Tracker Red is a type of lysosomal red fluorescent probe with weak alkaline labeling that can selectively stay in slightly acidic lysosomes, thus allowing lysosomal-specific staining. The fluorescence intensity increased under acidic conditions and decreased under alkaline conditions. Fluorescence microscopy showed that there was a large amount of red fluorescent spots in the cytoplasm of untreated cells, while the fluorescence density was significantly decreased after exposure to GO (Fig. 5a). With increasing GO concentration, the intracellular fluorescence intensity decreased, and the red fluorescence was the weakest at 80 μg/mL. The results suggest that GO also has a concentration-dependent effect on the acidity of lysosomes. Flow cytometry was performed to quantitatively analyze the red fluorescence intensity (Fig. 5b, c). The results showed that the cells in the control group exhibited the highest fluorescence strength. With the increase in GO dosage, the fluorescence intensity of cells decreased, and the curve gradually shifted to the left, which further suggested that GO affected the acidity of the lysosomal cavity, resulting in a certain degree of alkalization in a concentration-dependent manner. The results of the cathepsin B activity test revealed that cathepsin B activity was obviously lower in cells exposed to 80 μg/mL GO than in untreated cells (Fig. 5d). With the increase in GO concentration from 40 to 80 μg/mL, cathepsin B activity decreased in a concentration-dependent manner.

### GO induced apoptosis in F98 cells

**Fig. 6:**
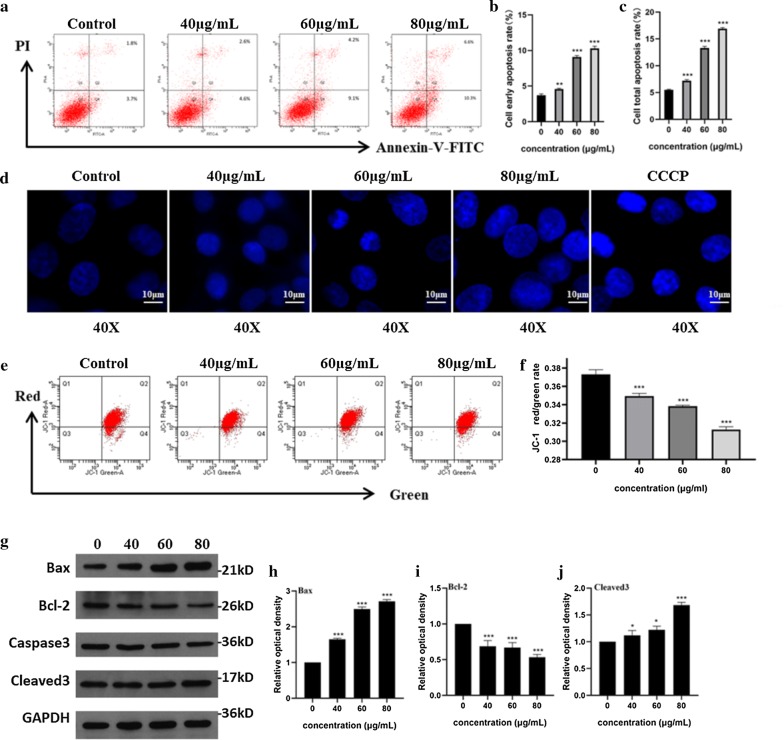
GO caused caspase-3-mediated apoptosis in a concentration-dependent manner. F98 cells were exposed to GO (40, 60 and 80 μg/mL) for 24 h. **a**–**c** The apoptosis was detected by AnnexinV-FITC-PI Apoptosis Kit. The early apoptosis rate (Q4) were located in the right lower quadrant and the total apoptosis rate (Q2 + Q4) are the sum of the right upper and the right lower quadrants. The column below shows the quantification of early and total apoptosis rates. **d** Before DAPI staining, CCCP, a kind of apoptosis inducer, was added as positive control to compare the changes of nucleus; **e**, **f** GO-exposed cells were dyed with a fluorescent carbocyanine dyestuff JC-1 to investigate the effects of GO on MMP by detecting the ratio of red and green fluorescence in flow cytometry. **g** The expression of Bax (**h**), Bcl-2 (**i**), cleaved 3 (**j**) in GO-exposed cells were identified by western blots. The column on the right showed the quantification of gray-scale value of straps through Imagine J analysis. All datas expressed as mean ± SD in three independent experiment results. *p < 0.05, **p < 0.01 and ***p < 0.001 vs. control group

Annexin V-FITC-PI double staining (Fig. 6a) showed that compared with untreated cells, the early apoptotic rate (Fig. 6b) and total apoptotic rate (Fig. 6c) of cells exposed to 80 μg/mL GO were 10.3% and 16.9%, respectively, which were higher than those exposed to 60 μg/mL and 40 μg/mL GO, indicating that GO caused dose-dependent apoptosis. DAPI staining was also used to observe changes in the nuclei during apoptosis (Fig. 6d). Fluorescence microscopy showed that the nuclei of the control group were uniformly colored and the fluorescence intensity was darker. The CCCP treatment group, as a positive control group, exhibited apoptotic cells with obviously enhanced fluorescent spots or plaques in the nuclei after staining. In the experimental group, with the increase in GO concentration from 40 to 80 μg/mL, the chromatic brightness of the nuclei increased [38, 39], and the fluorescence intensity inside the nuclei was uneven, with strong bright spots. The MMP detection kit is an ideal fluorescence probe that is extensively used to assay early cell apoptosis using the cationic fluorescent indicator JC-1. Red fluorescence can be produced when the MMP is high, and green fluorescence can be produced when the MMP is low. The red/green ratio can be generally used to assess the degree of mitochondrial depolarization. In this study, compared with control cells, the MMP assay showed that the red/green fluorescence ratio decreased significantly in GO-treated cells, and higher GO concentrations led to a more obviously decreased ratio (Fig. 6e, f). Studies have shown that MMP destruction is one of the earliest events in the process of cell apoptosis cascade reactions. Once MMP destruction occurs, apoptosis is irreversible [40]. The results showed that GO could reduce the red/green fluorescence ratio in a dose-dependent manner, which indicated that a loss of MMP and GO-induced concentration-dependent apoptosis occurred in F98 cells.

Western blots were used to observe the changes in related signaling pathways in the process of apoptosis. The results (Fig. 6g) showed that the proapoptotic proteins Bax (Fig. 6h) and cleaved caspase-3 (Fig. 6j) were upregulated and that the anti-apoptotic protein Bcl-2 (Fig. 6i) was downregulated after GO treatment in a concentration-dependent manner. Statistical analysis of the density of bands showed that the changes in histone levels in GO-treated cells were extensively different from those in untreated cells. Bax and Bcl-2 proteins are mainly located upstream of irreversible cell damage and play a role at the mitochondrial level. Thus, we suggested that GO can induce caspase-3-mediated apoptosis in F98 cells by endogenous signaling pathways.

## Discussion

It is noteworthy that the initial particle size of NPs often does not represent the real situation of their biomedical application. Compared with macromaterials, which are on the order of microns, nanomaterials belong to typical mesoscopic systems that have special effects, such as quantum size effects, surface and boundary effects, small-size effects and macroscopic quantum tunneling effects, and therefore, they more easily agglomerate when dispersed in solution. The average hydrodynamic size and zeta potential of GO detected by dynamic light scattering in pure water and cell culture medium are exhibited in Table 1. The hydrodynamic size in the cell medium was higher than that in pure water. A possible reason is that GO can combine with proteins, antibiotic, and other biomolecules in biological fluids leading to the formation of "nanoparticle-protein corona" [41]. Even though FBS protein in cell culture medium can slightly improve the dispersion stability of GO [42, 43], GO showed to be very stable in pure water, indicating GO was more stable in pure water than in culture medium and less likely to cause aggregation. The GO in pure water exhibited a negative charge (− 29.66 ± 0.79 mV), while the GO in complete culture medium showed a decreased negative charge of − 11.90 ± 1.06 mV. The results also indicated that GO is more stable in pure water. It is worth noting that before DLS characterization of GO, adequately ultrasonic treatment is extremely important to get a uniform suspension of GO.

**Table 1:** Zeta potential and hydrodynamic diameter of GO samples

GO samples	Hydrodynamic size (nm)	Zeta potential (mV)
In distilled water	409.18 ± 10.80	− 29.66 ± 0.79
In culture medium	747.02 ± 18.5	− 11.90 ± 1.06

Data are the mean ± SD of three independent experiments

In this study, we investigated the cell viability of F98 cells exposed to different concentrations of GO by CCK-8 detection and LDH test, whose result showed the concentration- and time-dependent cytotoxicity. Research reported that GO in a low concentration of 10 µg/mL up-regulates the homeostatic functions of primary astrocytes and modulates astrocyte-to-neuron communication without affecting viability [44]. It is expected that GO can cause cell death or increase cell viability depending on the cell line and the dosage [45, 46]. Notably, the cell viability assays revealed that F98 cells exhibited greater sensitivity to the LDH kit than to CCK-8 detection. As mentioned above, the material characterization by TEM showed that GO was flaky with sharp and irregular edges (Fig. 1b), which was helpful for penetrating the lipid bilayer and "inserting" into the cell. It has been reported that GO can influence the ultrastructure of the plasma membrane of different cells, resulting in a loss of plasma membrane integrity since various concentrations of GO dose-dependently increase the leakage of LDH [47, 48]. The sharp edge of the two-dimensional GO nanosheets can easily lead to abnormal cell membrane stretching and cytotoxicity. Similarly, GO caused concentration- and time-dependent cytotoxicity in cells due to damage to the plasma membrane, which may be relevant to the intense physical interaction between GO nanosheets and the phospholipid bilayer [49]. The surface of GO is also flexible and foldable, which gives it Tulle-like properties in biological medium, allowing it to easily accumulate or adhere to the cell membrane [50]. GO nanosheets adhere to and wrap around the cell membrane, can become inserted into the phospholipid bilayer, or interact at the cellular level to enter the cell [51]. SEM and TEM observations further confirmed that GO interacts with the cell membrane to induce cytotoxicity, which was consistent with the LDH test.

As clearly highlighted in previous studies, the possible toxic mechanisms of graphene-family nanomaterials include physical damage, ROS production leading to oxidative stress, mitochondrial damage, DNA damage, inflammatory response, apoptosis, autophagy and so on [52]. While some physicochemical properties and the toxicological effects of GO have been reported, the underlying toxicity and specific mechanisms of GO remain unclear and need to be further evaluated. In this study, the subsequent observation of autophagy using TEM and immunofluorescence staining assays revealed several interesting findings. First, the enhancement of fluorescence intensity may not necessarily represent the enhancement of autophagic activity but may also reflect hindrance of the autophagic lysosome degradation pathway [53]. It is well known that the transformation of LC3I into LC3II is a hallmark of autophagy. P62 protein is another autophagic marker protein besides LC3II/I. The expression level of p62 is often used to evaluate the degradation activity of autophagic lysosomes [54]. Accordingly, we further verified the expression of autophagy substrates of LC3 and p62 by Western blotting to assess the pathway of autophagic lysosome degradation. Combined with the results of cellular immunofluorescence and TEM, we suggest that GO activates autophagy and induces the transformation of LC3I to LC3II. At the same time, to a certain extent, GO impairs the degradation function of autophagy flux, resulting in a reduction in ubiquitination substrate degradation mediated by p62, which leads to the abnormal accumulation of p62 [55–57]. Studies have shown that GO has adverse effects on mouse peritoneal macrophages with autophagosome accumulation and the conversion of LC3-I to LC3-II. The degradation of the autophagic substrate p62 protein was also inhibited. Further analyses on lysosomes showed that the accumulation of GO in macrophage lysosomes led to the instability of lysosomal membrane, indicating reduced autophagic degradation [58]. These results of abnormal autophagy flux are consistent with our results. However, the specific signaling pathways involved in this process have not been discussed, and we designed further experiments to explore.

Autophagy is regulated by a variety of signaling pathways. Mammalian target of rapamycin (mTOR) is a relatively conservative protein kinase of the phosphatidylinositol 3-kinase (PtdIns3K)-related family. Many studies have suggested a leading status for mTOR in autophagy [53, 59, 60]. Phosphatidylinositide 3-kinases (PI3K) and serine/threonine kinase (Akt) are upstream of mTOR, which could lead to the deactivation of mTOR by phosphorylation and trigger autophagy. In this study, the expression of PI3K/Akt/mTOR, a main upstream signaling pathway of autophagy, was further studied by Western blot analysis, and the results suggested that GO-induced autophagy was activated by suppressing the PI3K/Akt/mTOR signaling pathway. Previous studies have shown that GO-activated autophagy does not involve the mTOR pathway but depends on activation of the PtdIns3K and MEK/ERK1/2 signaling pathways [61]. A recent study also suggested that downregulation of PI3K/Akt/mTOR signaling pathways leads to GO-induced autophagy [31].

Extensive studies have established that impairment of autophagy flux and lysosomal dysfunction are emerging mechanisms of NP cytotoxicity. For instance, graphite carbon nanofibers could impair autophagy flux via lysosomal dysfunction, which leads to autophagosome accumulation [56]. Zinc oxide NP-induced autophagic flux disorder and lysosomal malfunction were closely correlated with toxicity [62]. Accordingly, this study further examined whether the situation and function of lysosomes were impaired to explore the basis of autophagic dysfunction. The intraluminal pH value of lysosomes (pH = 4.5–5.5) is one of the most important factors affecting lysosomal physiological function [63, 64]. Studies have shown that NPs can induce lysosome dysfunction by affecting acidity [65]. Therefore, it is necessary to monitor the pH of lysosomes for studying lysosome degradation function. Cathepsin B, a cysteine proteolytic enzyme, is active in the pH range of 3.0–7.0, but it can be irreversibly inactivated under alkaline conditions, which plays an essential role in lysosomal degradation of substrates. The results of this study suggest that the alkalization of lysosomes can lead to a decrease in cathepsin B activity and dysfunction of autophagic flux, thus further blocking degradation of the autophagy substrate p62 and leading to its accumulation.

The main function of the lysosome is digestion; lysosomes not only participate in the development of autophagic flux but are also the most important digestive organ in cells. The normal function of lysosomes is very important for maintaining the stability of the intracellular environment, resisting harmful stimulation and renewing intracellular components. Damage to lysosomal function will have adverse effects on cell viability and even lead to apoptosis [63, 66, 67]. Previous studies have shown that graphene and GO can induce the loss of MMP and the increase of ROS, which trigger MAPKs- and TGF-β related signaling pathways, and activate caspase-3 in a mitochondrial dependent manner, thus initiating the execution of apoptosis [68, 69]. In this study, multiple biochemical detection methods were used to observe the occurrence of apoptosis from many aspects after lysosomal dysfunction. Apoptosis is a type of programmed cell death. There are two major pathways involved in this process: the exogenous signaling pathway triggered by the death receptor and the endogenous signaling pathway initiated by mitochondria. There are intricate relationships between the two pathways that together regulate the apoptosis process of cells. Caspase, known as apoptosis protease, plays an irreplaceable role in both apoptosis pathways. It is well known that caspase-3 is a vital terminal shear during apoptosis [70]. Cleaved caspase-3 can degrade many kinds of cell proteins and is responsible for the charge of the morphological changes and DNA breakage in the process of apoptosis. The Bcl-2 gene enzyme e is the first to be found and confirmed to play a crucial role in monitoring apoptosis. Its expression products together constitute the Bcl-2 family. Some members of the Bcl-2 family, such as Bcl-2, show resistance to apoptosis, whereas others, such as Bax, show promotion of apoptosis [71, 72]. The results of this study indicated caspase-3 mediated apoptosis after GO exposure.

**Fig. 7:**
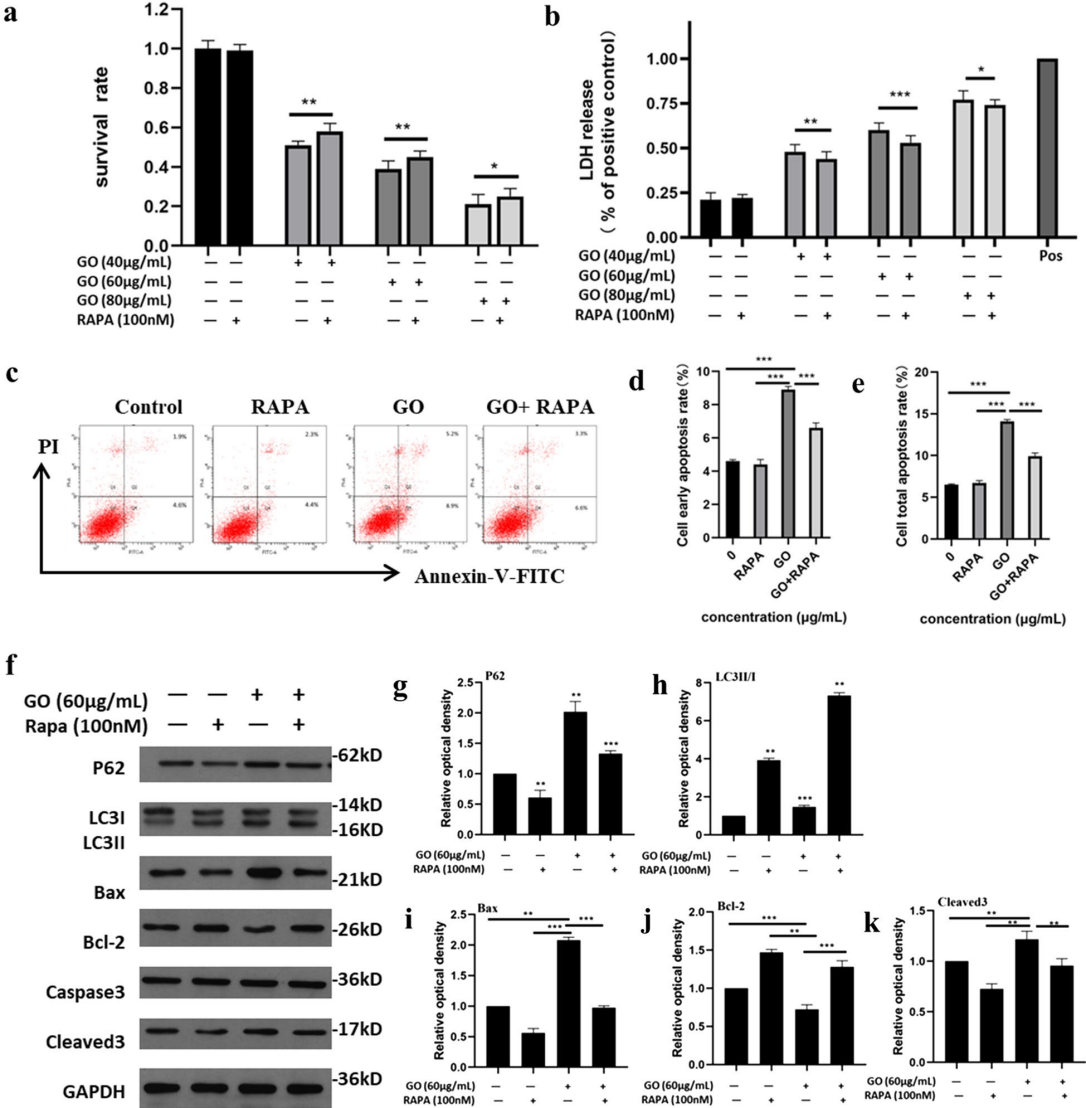
Verification of P62-mediated cytotoxic response. **a**, **b** F98 Cells were exposed to GO (40, 60, 80 μg/mL) with or without treatment of rapamycin (8 nM) for 24 h. The cell viability was analyzed using CCK-8 and LDH kits, respectively. **c**–**e** F98 cells were treated with 60 μg/mL GO with or without treatment of rapamycin (8 nM) for 24 h to investigate the cell apoptosis by Annexin V-FTIC-PI apoptosis kit. The column on the right exhibited the quantification of flow cytometry results. **f** Similarly, GO-treated cells were incubated with or without rapamycin for 24 h. The expression of p62 (**g**), LC3II/I (**h**), Bax (**i**), Bcl-2 (**j**), cleaved 3 (**k**) were detected by western blots. The column on the right showed the quantification of gray-scale value of straps through Imagine J analysis. All datas expressed as mean ± SD in three independent experiment results. *p < 0.05, **p < 0.01 and ***p < 0.001 vs. control group

Through the above experiments, we suggest that GO could induce the blocking of autophagic flux through repairing the physiological function of lysosomes and even resulting in apoptosis after internalization into cells. Notably, blockade of autophagic flux leads to excessive accumulation of p62 protein. On the one hand, it causes lysosome overload, aggravates lysosome membrane damage and releases more hydrolases to cause cell damage. On the other hand, p62, as a cross-linking protein of the autophagy and apoptotic pathway, may also participate in the process of apoptosis [54, 73]. Autophagy is a complex stress process involving many steps and dozens of proteins. The regulation of different proteins in the autophagy process through drug intervention will have complex and variable effects on the autophagy process. Rapamycin, a classical autophagic activator, can effectively promote the development of autophagic flux by inhibiting the function of mTOR, which is widely used in autophagy research. As shown in Fig. 7a, treatment with 100 nM rapamycin alone caused no significant decrease in cellular viability compared with the control group, indicating that rapamycin in this dose had good biocompatibility. At the same time, when cells were treated with GO in the presence of rapamycin, the GO-induced cell viability decline was significantly alleviated, and the salvage effect for different concentrations of GO treatment is shown. The LDH test results (Fig. 7b) are consistent with the CCK-8 assay. Similarly, rapamycin could also alleviate GO-induced apoptosis according to the Annexin V-FITC-PI double staining test (Fig. 7c). Rapamycin combined with GO significantly reduced GO-induced apoptosis, including reducing the early apoptotic rate (Fig. 7d) and the total apoptotic rate (Fig. 7e), which can be reflected when the concentration of GO is 40, 60, and 80 μg/mL. Statistical analysis also suggested that there was a marked divergence between the two groups. Western blots showed that the level of LC3 II/I was upregulated and the level of p62 was downregulated significantly compared with the control group (Fig. 7f–h).

These results suggest that rapamycin treatment alone could effectively promote the development of autophagic flux. In addition, when cells were treated with GO in the presence of rapamycin, the proapoptotic proteins Bax and cleaved caspase-3 were downregulated, while the anti-apoptotic protein Bcl-2 was upregulated (Fig. 7i–k). These results suggest that rapamycin could promote the development of autophagic flux and alleviate the abnormal accumulation of p62 in GO-treated cells. Furthermore, rapamycin could increase the viability of cells and showed certain rescue effects against apoptosis. The abnormal accumulation of p62 plays an important role in GO-induced cytotoxicity in F98 cells, and alleviating the abnormal accumulation of p62 in F98 cells can alter GO-induced cytotoxicity.

## Conclusion

In the present study, we reported the interaction of GO with the rat astroglioma-derived F98 cell line accompanied by interlink between autophagy, lysosomes and apoptosis. The results indicate that the exposure of F98 cells to GO can elicit concentration- and time-dependent toxicological effects. GO-treated cells exhibited autophagy activation by inhibiting the PI3K/Akt/mTOR signaling pathway. Simultaneously, GO can induce the dysfunction of lysosomes via alkalizing lysosomes and then block autophagic flux, resulting in the abnormal accumulation of autophagic substrate p62 protein. Abnormal accumulation of p62 protein can induce capase-3-mediated apoptosis. Rapamycin, an autophagy activator, can promote autophagic flux development and alleviate the occurrence of GO-induced cytotoxicity. This study sheds light on the lack of effect of GO on astrocytes and provides insights into the safe application of graphene-family NPs in the CNS. Moreover, it is clear that astrocytes exposed to GO can downregulate the PI3K/Akt/mTOR signaling pathway to activate autophagy; however, the pathway of upregulated autophagy activation remains unclear, and further research is needed in the future.

## Data Availability

The datasets used and/or analyzed in the current study are available from the corresponding author on reasonable request.

## Abbreviations

Akt: Serine/threonine kinase
AFM: Atomic force microscope
BBB: Blood–brain barrier
BCA: Bicinchoninic acid
CCCP: Carbonyl cyanide 3-chlorophenylhydrazone; carbonyl cyanide m-chlorophenylhydrazone
CCK-8: Cell counting kit-8
CNS: Central nervous system
DAPI: 2-(4-Amidinophenyl)-6-indolecarbamidine dihydrochloride
DMEM: Dulbecco’s modified Eagle’s medium
FBS: Fetal bovine serum
FTIR: Fourier-transform infrared spectroscopy
GAPDH: Glyceraldehyde-3-phosphate dehydrogenase
GO: Graphene oxide
HRP: Horseradish peroxidase-conjugated
LC3: Microtubule-associated protein 1 light chain 3
LC3I: Cytosolic LC3
LC3II: Phosphatidylethanolamine (PE)-conjugated form
LDH: Lactate dehydrogenase
MMP: Mitochondrial membrane potential
NPs: Nanoparticles
PI3K: Phosphatidylinositide 3-kinases
PVDF: Polyvinylidene fluoride
SEM: Scanning electron microscopy
SD: Standard deviation
TEM: Transmission electron microscopy
XPS: X-ray photoelectron spectroscopy
